# Brugada ECG Pattern Unmasked by IV Flecainide in an Individual with Idiopathic Fascicular Ventricular Tachycardia

**DOI:** 10.1016/s0972-6292(16)30590-3

**Published:** 2013-01-01

**Authors:** Andrew R Gavin, Glenn D Young, Andrew D McGavigan

**Affiliations:** 1 Department of Cardiovascular Medicine, Flinders Medical Centre; 2 Department of Medicine, University of Adelaide; 3 Faculty of Medicine, Flinders University of South Australia

**Keywords:** monomorphic ventricular tachycardia, Brugada, fascicular, idiopathic

## Abstract

A 45-year old man presents with stable monomorphic ventricular tachycardia. He had previously been diagnosed with idiopathic fascicular ventricular tachycardia. Intravenous flecainide results in termination of his tachycardia but unmasks a latent type 1 Brugada ECG pattern not seen on his resting ECG. We discuss his subsequent management and the need to consider an alternative diagnosis in individuals with a Brugada type ECG pattern who present with stable monomorphic ventricular tachycardia.

## Case Report

**Figure 1:**
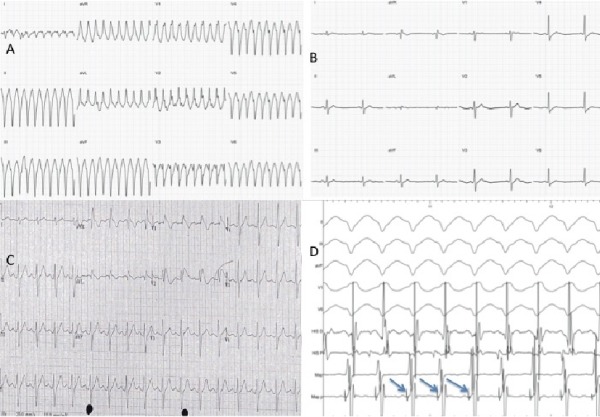
(A) Classical left posterior fascicular VT with right bundle branch block pattern and superior axis. (B) Resting ECG with no ST elevation. (C)Type 1 Brugada ECG pattern with coved ST elevation and J point elevation in leads V1 and V2 following administration of intravenous flecainide. (D) Recordings taken at time of EPS during VT. Sweep speed of 100mmm/sec. Displayed (from top) are 4 leads from ECG, 2 intracardiacelectrograms from quadripolar catheter at the His position and 2 recordings from mapping catheter (Map) in the region of the posterior fascicle. A high frequency short duration Purkinje potential (arrows) is seen preceding the onset of the QRS.

A 45-year old man presented with sudden onset palpitations whilst exercising. There was no associated hemodynamic compromise. Electrocardiogram (ECG) showed monomorphic ventricular tachycardia (VT) with a right bundle branch block pattern and a superior axis (Figure 1A).

He gave a history of 3 prior episodes over the preceding 20 years and had seen an electrophysiologist previously. There was no history of syncope and no family history of sudden cardiac death. Resting ECG was within normal limits (Figure 1B) and echocardiography demonstrated a structurally normal heart. At that time, a diagnosis of idiopathic fascicular VT was made and after discussion the patient elected not to undergo an electrophysiology study (EPS).

On this occasion he was treated in the Emergency Department with IV flecainide at the instruction of his treating electrophysiologist. This resulted in termination of tachycardia with restoration of sinus rhythm but unmasked a latent type 1 Brugada ECG pattern (Figure 1C), not seen on his resting ECG (Figure 1B). In view of his history of ventricular arrhythmia and a type I Brugada ECG pattern he was referred by the admitting team for consideration for an implantable defibrillator (ICD).

**Figure 2:**
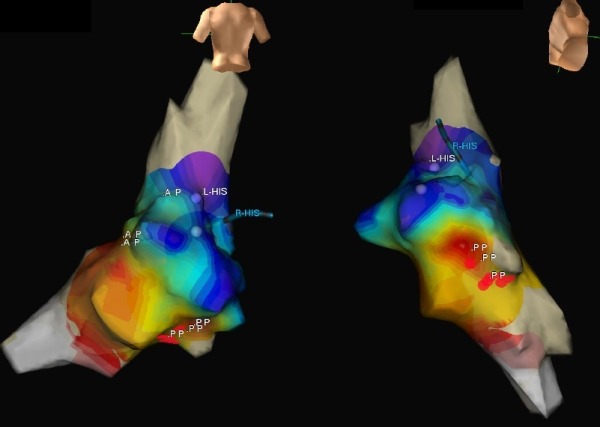
3D reconstruction and activation map of left the ventricle using the EnSite Velocity System. PA view is on the left and an extreme RAO view with inferior tilt on the right. His potentials recorded from right and left sides of septum are marked as L-His and R-His respectively. Sites with purkinje potentials representing the anterior and posterior fascicles of the left bundle branch are marked as AP and PP respectively. The PP potentials lie close to the red dots which indicate the site of successful ablation. The successful ablation site is proximal to the earliest activation (displayed in white). This is because although the circuit is within the posterior fascicle, the activation times were calculated from near-field ventricular electrograms and not timing of the Purkinje potentials. As such, the earliest ventricular activation is displayed at the ventricular insertion of the Purkinje fibres.

However, following discussion with the electrophysiology service, a diagnostic EPS with a view to ablation of his idiopathic fascicular VT was recommended. Following administration of intravenous isoproterenol, monomorphic VT (CL 260ms) was easily induced on multiple occasions with programmed ventricular stimulation. This VT had an identical morphology to that seen on arrival at the Emergency Department. An externally irrigated ablation catheter (Cool Path Duo, St Jude Medical, St Paul, MN, USA) was advanced retrogradely to the left ventricle and a 3 dimensional (3D) left ventricular geometry and activation map was created using Ensite Velocity (St Jude Medical, St Paul, MN, USA) (Figure 2).

Earliest activation of the ventricle was seen at the insertion of the posterior fascicle with centrifugal activation of the rest of the chamber. The catheter was drawn back along the inferior septum to the region of the posterior fascicle until a discrete Purkinje potential could be identified preceding each ventricular electrogram (Figure 1D). Ablation was performed (maximum 40W) in the region of the posterior fascicle (Figure 2). Following ablation no polymorphic or monomorphic VT or ventricular fibrillation was inducible with programmed stimulation and up to 3 extra beats either in the baseline state or with isoproterenol.

## Discussion

We present a case of monomorphic VT in a patient with a type 1 Brugada ECG pattern following injection of intravenous flecainide. This raises the possibility of either monomorphic VT as part of the Brugada syndrome or of a latent Brugada ECG pattern in an individual with idiopathic fascicular VT. The common form of idiopathic fascicular VT is characterized by a relatively narrow QRS width with right bundle branch block morphology and a superior axis [1]. It commonly presents in the second to fourth decade, is usually paroxysmal and is associated with a structurally normal heart. It is commoner in males and has a good prognosis. It can be induced by exercise, atrial and ventricular premature beats and atrial and ventricular pacing, and can be terminated using verapamil. The tachycardia is thought to be due to a reentrant circuit close to the posterior fascicle of the left bundle branch. In this case the administration of intravenous flecainide presumably slowed a critical part of the circuit sufficiently to produce block thereby resulting in termination of the tachycardia and restoration of sinus rhythm. At EPS, high frequency Purkinje potentials are seen preceding the site of earliest ventricular activation during VT. Catheter ablation is the preferred treatment choice for fascicular VT with a success rate of greater than 90%.

Brugada syndrome was first described in 1992 and is characterized by episodes of syncope or unexpected sudden death in patients with structurally normal hearts [2]. It classically presents with polymorphic VT/VF. Whilst a Brugada ECG refers to the manifestation of a type 1 ST-segment elevation, Brugada syndrome is only definitively diagnosed when these changes are observed in conjunction with one or more of the following: documented VF, polymorphic VT, a family history of sudden cardiac death below 45 years of age, coved-type ECG changes in family members, inducibility of polymorphic VT with programmed electrical stimulation, syncope or nocturnal agonal respiration [3].

In this case although he undoubtedly has a type 1 Brugada ECG pattern he has no other features to indicate he has Brugada syndrome. The ease of inducibility of VT, the intra-cardiac EGMs, the activation map and the site of successful ablation all support a diagnosis of idiopathic fascicular VT as the likely cause for his tachycardia.

While monomorphic VT has previously been reported in individuals with Brugada Syndrome [4] it is extremely rare. An ICD in this gentleman would have been inappropriate putting him a risk of potential defibrillation, with its associated morbidity and mortality risks, for non-life threatening arrhythmias.

This case highlights the importance of excluding other, potentially treatable causes of idiopathic VT prior to ICD implantation. This is especially true in those with a Brugada type ECG pattern who are considered at low risk of sudden cardiac death, where appropriate ablative strategies can negate the need for a defibrillator.
